# Transcriptomic analysis of drought stress responses of sea buckthorn (*Hippophae rhamnoides*subsp. sinensis) by RNA-Seq

**DOI:** 10.1371/journal.pone.0202213

**Published:** 2018-08-13

**Authors:** Guisheng Ye, Yuhua Ma, Zhipeng Feng, Xiaofen Zhang

**Affiliations:** 1 State Key Laboratory of Plateau Ecology and Agriculture, Qinghai University, Xining, China; 2 College of Agriculture and Animal Husbandry, Qinghai University, Xining, China; Jawaharlal Nehru University, INDIA

## Abstract

Sea buckthorn is one of the most important eco-economic tree species in China due to its ability to grow and produce acceptable yields under limited water and fertilizer availability. In this study, the differentially expressed genes under drought stress (DS) of sea buckthorn were identified and compared with control (CK) by RNA-Seq. A total of 122,803 unigenes were identified in sea buckthorn, and 70,025 unigenes significantly matched a sequence in at least one of the seven databases. A total of 24,060 (19.59%) unigenes can be assigned to 19 KEGG pathways, and 1,644 unigenes were differentially expressed between DS and CK, of which 519 unigenes were up-regulated and 1,125 unigenes down-regulated. Of the 47 significantly enriched GO terms, 14, 7 and 26 items were related to BP, CC and MF, respectively. KEGG enrichment analysis showed 398 DEGs involved in 97 different pathways, of which 119 DEGs were up-regulated and 279 DEGs were down-regulated under drought stress. In addition, we found 4438 transcriptor factors (TFs) in sea buckthorn, of which 100 were differentially expressed between DS and CK. These results lay a first foundation for further investigations of the very specific functions of these unigenes in sea buckthorn in response to drought stress.

## Introduction

Globally, arid and semi-arid area amounts to 60.9 million km^2^, covering 41.3% of the Earth’s total area [1]. There are 14 million km^2^ of cultivated area around the world, of which 6 million km^2^ belong to arid and semi-arid regions; the total arid and semi-arid area in China accounts for 50% of the national territory [2]. Plants in these areas are frequently subjected to drought stress during their life cycle, and drought reduces plant biomass and grain yield [3]. Statistical data have shown that the total loss caused by meteorological disasters accounts for approximately 85%, of which drought accounts for approximately 50% [4]. Since the 1970s, droughts have become longer and more severe across the globe, and this trend will continue in the future [5,6]. Social problems such as decreased production of agriculture, food shortage, and malnutrition linked to droughts will become more frequent and more severe [7]. This situation will be exacerbated by increasing global population, urbanization and industrialization of occupied arable land, land degradation and desertification [8,9]. Climate change will continue to limit water availability through increased temperature and the frequency and/or severity of droughts [10–12]. Therefore, agricultural productivity faces a greater challenge in fighting drought stress. Drought is regarded as one of the most damaging natural disasters in agricultural production, and it is a hot area of research [13].

It is impossible to expand farm land area to satisfy the demand of plant-based food, and adverse environmental factors such as frequent drought or other abiotic stress have prompted us to cultivate new varieties with high stress-resistant capacity, which can maintain stable production under a harsh environment.

To increase the productivity and stress resistance of plants, past efforts used conventional breeding (e.g., introduction and domestication, hybrid breeding, selection breeding). However, these breeding approaches have had little success in improving the target traits of plants and are constrained by genetic resistance, reproductive barriers, and long generation times that limit the transfer of favorable alleles from diverse genetic resources [14–15].

In recent years, with the rapid development of high-coverage whole genome sequences, transcriptomics and functional genomics, especially sequencing technology and genetic engineering, transgenic approaches have emerged as an important and effective path to provide target traits, as we anticipate the introduction of target genes derived from the same plant species or from another genus [16]. Many useful genes are used as candidate sequences coupled to a transgenic approach, and based on the current findings, molecular breeding shows great potential to dramatically enhance the stress tolerance of crops and promote growth and development under harsh environments [7,17–19]. These developments are initiating a new technology revolution in crop resistive breeding research.

Although there significant progress has been made to enhance the drought stress resistance of crops using a transgenic approach and obtain transgenic lines, most have higher tolerance to drought stress, but many also show growth retardation and a yield also affected by drought stress [17,20,21]. Therefore, more efforts are needed to discover more effective genes from drought stress-resistant plants. We must thus unravel the molecular mechanisms by which plants perceive and transduce stress signals to cellular machinery to initiate responses and then identify key genes and pathways to engineer stress-tolerant crops.

Due to the high-speed development of sequencing techniques and genomics, transcriptomics, proteomics and many bioinformatics analysis tools, major progress has been made in decoding stress signaling pathways and the key genes involved in plant abiotic stress response [17]. To date, stress-related genes have been identified by RNA-Seq, and their expression modes under drought stress have already been clarified in many plants including maize [22], cotton [23], rice [24], soybean [25], and sorghum [26].

Sea buckthorn is a thorny, non-leguminous, nitrogen- fixing, deciduously perennial shrub that is widespread in Asia and Europe [27]. The berries of sea buckthorn contain abundant bioactive compounds, and because of its outstanding economic potential, sea buckthorn has attracted considerable attention from researchers around the world, mainly for its nutritional and medicinal value. Although many publications focus on sea buckthorn, few have been related to the mechanism of drought stress resistance and discovery of functional genes.

In recent years, some reports have assessed sea buckthorn based on next-generation sequencing, such as Fatima et al. (2012) [28], Chaudhary and Sharma. (2015) [29], Ghangal et al. (2013) [30], Sharma et al. (2016) [31] and Li et al. (2017) [32]. These studies were performed on sea buckthorn using next-generation sequencing with different aims and objectives, but there is no report on transcriptional analysis in sea buckthorn under drought stress. As a result, the complex molecular mechanisms, signaling perception and transduction in response to drought stress in sea buckthorn are not well-understood.

In this study, based on genome-wide transcriptome profiling, we employ RNA-Seq to understand the response of sea buckthorn to drought stress. The result will enable us to understand the molecular responses of sea buckthorn against drought stress, and the identified genes in this paper can be used as candidate genes for engineering plants to enhance their stress tolerance under drought stress.

## Materials and methods

### Plant material and stress treatment

*Hippophae rhamnoides* subsp. sinensis (sea buckthorn) is widely distributed in Qinghai Province, China. The field study in this work did not involve endangered or protected species, and no specific permissions were required for the field sampling. The berries of sea buckthorn were collected from Datong county in Qinghai province (37°14'45"N and 101°30'15"E, altitude 2920 m). The seeds were surface-sterilized with potassium permanganate and incubated in water for 48 h at 40°C for germination, then sowed in 30 cm (height) × 25 cm (diameter) pots (peat:soil:perlite mixture = 40:30:30 (vol%)). The pots were covered with plastic film until 1/3 of them sprouted. All plants were kept in a greenhouse (20–28°C, 60–70% rH). The seedlings were watered every third day with tap water (100 mL/pot) and fertilized every 2 weeks with Hoagland’s solution. The 5-month-old seedlings were selected for our experiment. The seedlings were randomly divided into 2 groups. The first group was watered as before (control, CK), while the second group received no further watering (drought stress treatment, DS). The drought stress lasted for 2 weeks, and then, all groups were collected separately and immediately frozen in liquid nitrogen and stored at -80°C. The experiment was conducted at the State Key Laboratory of Plateau Ecology and Agriculture (Qinghai University) from March to August 2016.

### RNA extraction and quality control

Total RNA was extracted from the leaves of sea buckthorn using an EASYspin Plus plant RNA kit (Aidlab Biotech, China) following the manufacturer's protocol. RNA degradation and contamination were monitored by 1% agarose gels. The quality and purity of RNA were assessed by determining the absorbance at 280, 260 and 230 nm using a NanoPhotometer (IMPLEN, CA, USA). RNA was only used when the OD260/280 was greater than 1.8. RNA concentration was measured using a Qubit® RNA Assay Kit in a Qubit® 2.0 Fluorometer (Life Technologies, CA, USA). RNA integrity was assessed using the RNA Nano 6000 Assay Kit of the Agilent Bioanalyzer 2100 system (Agilent Technologies, CA, USA). The total RNA was stored at -80°C for later use. Three biological replicates were used for our experiment.

### Library preparation and transcriptome sequencing

A total of 1.5 μg of RNA was used as the input material for RNA sample preparations. Sequencing libraries were generated using a NEBNext® Ultra™ RNA Library Prep Kit for Illumina® (NEB, USA). The clustering of the index-coded samples was performed on a cBot Cluster Generation System using a TruSeq PE Cluster Kit v3-cBot-HS (Illumina) according to the manufacturer’s instructions. After cluster generation, the library preparations were sequenced on an Illumina HiSeq platform by Novogene (Beijing, China), and paired-end reads were generated.

Raw reads in the fastq format were first processed through in-house Perl scripts. Clean reads were obtained by removing reads containing adapter or ploy-N and discarding low-quality reads from raw reads. The Q20, Q30, GC-content and sequence duplication level of the clean reads were calculated. All clean reads were assembled with Trinity software using the default parameters (https://github.com/trinityrnaseq/trinityrnaseq/wiki) [33].

### Gene functional annotation

Gene function was annotated based on the following databases: NCBI non-redundant protein sequences (Nr), NCBI non-redundant nucleotide sequences (Nt), Protein family (Pfam), Clusters of Orthologous Groups of proteins (KOG/COG), SwissProt (a manually annotated and reviewed protein sequence database), KEGG Ortholog (KO) and Gene Ontology (GO). Transcription factors (TFs) were predicted using iTAK software[34].

### Differential gene expression analysis

Gene expression levels for each sample were estimated by the RSEM program[35]. Gene expression was calculated by the number of fragments per kilobase of transcript sequence per million base pairs sequenced (FPKM). The differentially expressed genes (DEGs) between CK and DS were identified using the DESeq R package (1.10.1)[36]. Adjusted P values were used to control the false discovery rate, and all the genes with an adjusted P-value (padj) < 0.05 were assigned as DEGs. GO enrichment analysis of the DEGs was implemented by GOseq R packages[37], and KOBAS[38] was used to test the statistical enrichment of DEGs in the KEGG pathways.

### Quantitative PCR analysis

To verify the reliability of the RNA-Seq data for sea buckthorn, 10 unigenes (S1 Table) were randomly selected for qRT-PCR analyses using a Power SYBR Premix Ex TaqTM II Kit (Perfect RealTime, Takara, Dalian, China) with a Bio-Rad CFX96 Real-Time PCR system (Bio-Rad, USA) according to the manufacturer’s instructions. β-actin gene was selected as an internal control. The relative expression was calculated by the delta-delta CT method and expressed as the fold change relative to expression in the null controls (expression = 1).

### Submitting RNA-Seq data

The RNA-Seq data for sea buckthorn were submitted to the NCBI Sequence Read Archive (SRA), and the accession numbers are as follows:

SRA: SRR7003894, SRR7003893, SRR7003896, SRR7003895, SRR7003892, SRR7003891 (SUB3892164)

BioProject: PRJNA449450 (SUB3883118)

BioSample: SAMN08895389: CK_1, SAMN08895390: CK_2, SAMN08895391: CK_3, SAMN08895392: XP_1, SAMN08895393: XP_2, SAMN08895394: XP_3 (SUB3881522) (Note: XP sample name corresponds to DS in the manuscript)

## Results

### RNA-Seq of sea buckthorn and de novo assembly

RNA-Seq of the six cDNA libraries (three repeats for CK and DS) resulted in 33.77 million raw reads, of which approximately 32.56 million clean reads were *de novo* assembled into contigs using Trinity software, ranging from 4.66 million to 5.93 million reads per library and more than 95% of reads exhibiting a quality score of Q20 (99% accuracy) (Table 1). The contigs were assembled into 122,803 unigenes with an average length of 1,151 bp and an N50 length of 1,890 bp. All unigenes were longer than 200 bp, and 38.06% (46,746 unigenes) of them were longer than 1,000 bp.

**Table 1:** Overview of sequencing and assembly.

Sample	Raw Reads (million)	Clean reads (million)	Clean bases (G)	Q20 (%)	Q30 (%)	N (%)	GC (%)
**CK-1**	5.61	5.42	8.13	96.84	92.27	1.75	41.82
**CK-2**	6.14	5.93	8.89	96.28	91.14	1.66	41.78
**CK-3**	5.66	5.47	8.2	96.73	92.06	1.74	41.74
**DS-1**	5.65	5.41	8.12	95.75	90.08	1.75	41.60
**DS-2**	5.87	5.67	8.5	96.48	91.57	1.73	41.10
**DS-3**	4.84	4.66	6.99	96.15	90.9	1.72	41.63
**Total/Mean**	33.77	32.56	48.83	96.37	91.34	1.725	41.61

### Functional annotation and classification of unigenes

**Fig 1:**
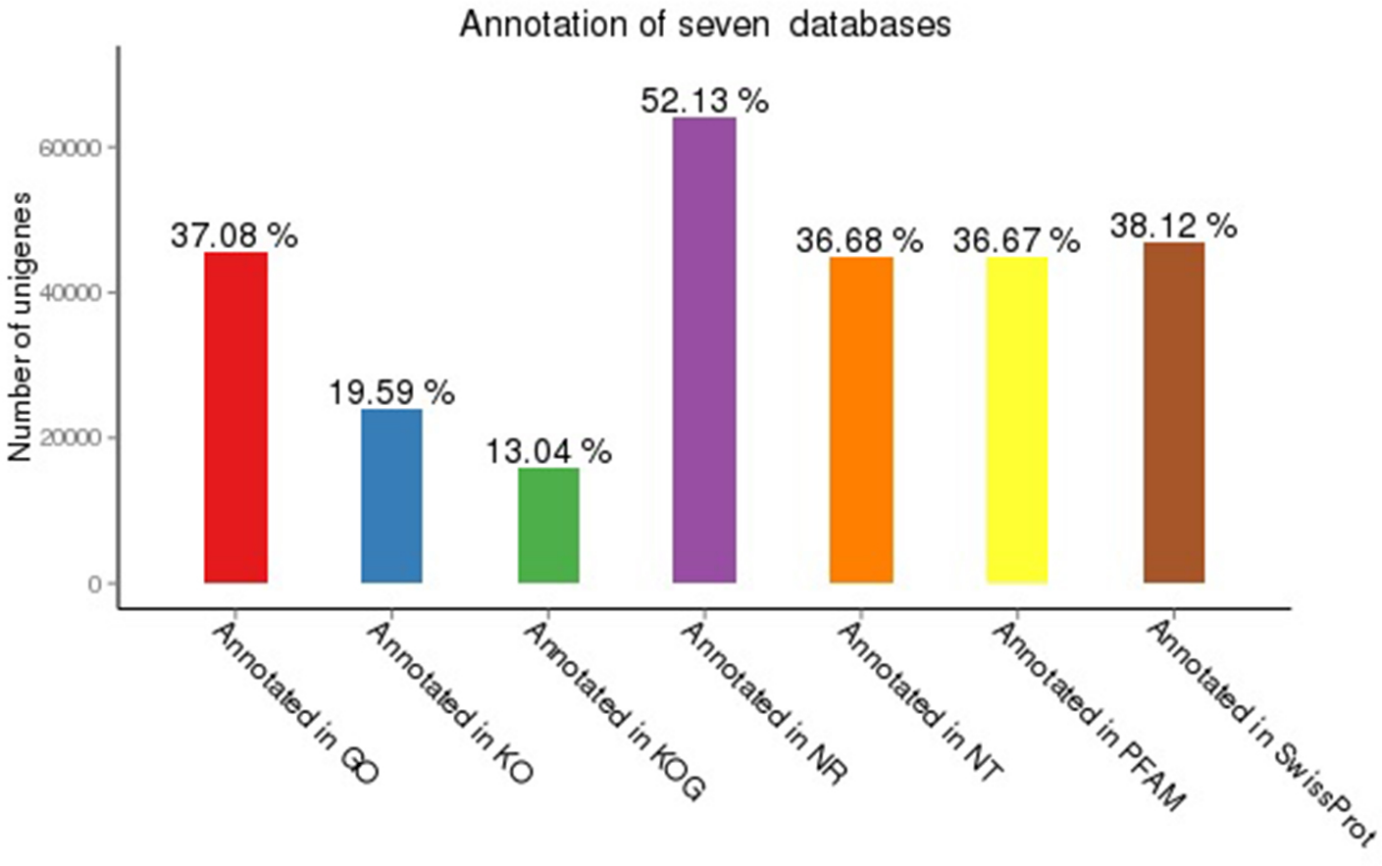
Unigenes matched in seven databases.

A total of 64,019 (52.13%), 45,046 (36.68%), 24,060 (19.59%), 46,817 (38.12%), 45,038 (36.67%), 45, 543(37.08%) and 16,019 (13.04%) unigenes had significant hits (E-value <10−5) in NR, NT, KO, SwissProt, PFAM, GO and KOG, respectively (Fig 1). Of the 122,803 high-quality unique sequences, 70,025 (57.02%) unigenes significantly matched a sequence in at least one of the seven databases, and 9,297 (7.57%) unigenes showed similarity to proteins in all seven databases. Compared with Ghangal’s results, only 41,340 (46.8%) among 88,297 transcripts were significant hits with the NCBI database; our annotation rate was higher than that from Ghangal et al 2013[30]. We also analyzed the common annotation rate of Ghangal’s[30] results with ours, the result showed that there have 35584 unigenes co-annotated in both research, it’s account about 29% and 40% of the total unigenes generated in the study respectively.

**Fig 2:**
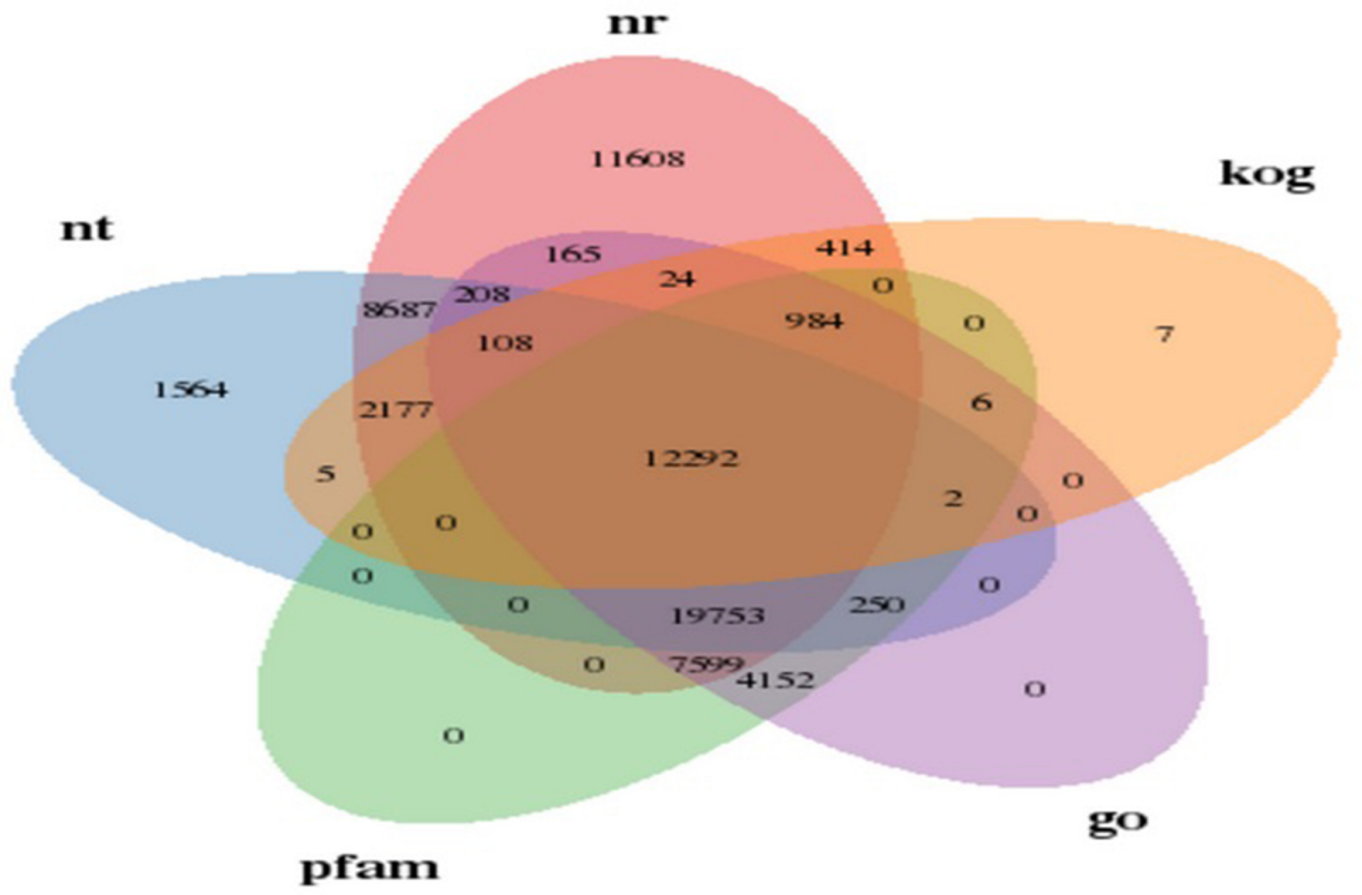
Venn diagram for differential BLAST results of sea buckthorn. Note: The number of unigenes annotated with 5 databases and the co-annotated gene number are shown.

The five main public databases (NT, NR, KOG, GO and PFAM) from seven databases were selected to draw a Venn diagram (Fig 2): the number of unigenes with significant motifs (E-value≤10^−5^) is shown at each intersection of the Venn diagram, in which 12,292 unigenes matched in all five databases.

**Fig 3:**
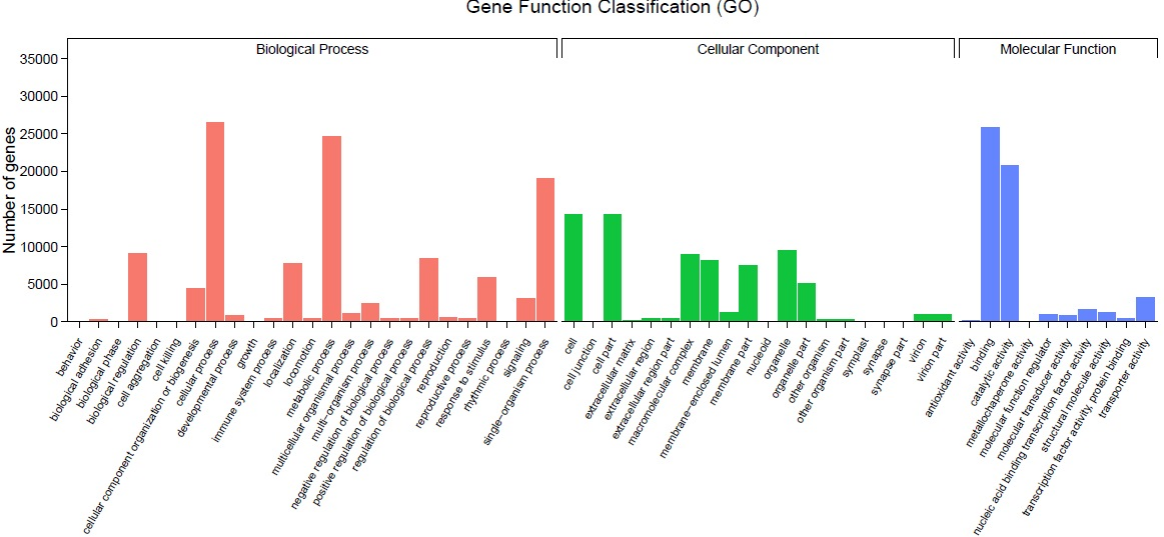
Functional classification of GO terms of sea buckthorn transcripts. Note: The number of genes in a specific sub-category within the main category is shown on the y-axis; the name of the sub-category is shown on the x-axis.

Based on the high-score BLASTx matches in the GO proteins database, a total of 45,543 unigenes were classified with Blast2GO [39] (E-value <10^−5^) and was assigned at least one GO term. As shown in Fig 3, the unigenes belonged to three main GO categories and 55 sub-categories, including biological processes (BP), with 25 main sub-categories (116,901 unigenes); cellular compartments (CC), with 20 main sub-categories (71,616 unigenes); and molecular functions (MF), with 10 main sub-categories (55,022 unigenes).

The largest sub-groups in the biological process category were cellular process (22.68%), metabolic process (21.08%), single-organism process (16.33%), biological regulation (7.82%) and regulation of biological process (7.26%). In the cellular component category, the largest subgroups were cell (19.94%), cell part (19.93%), organelle (13.30%), macromolecular complex (12.58%) and membrane (11.32%). In the molecular function category, the largest subgroups were binding and catalytic activity, which accounted for 47.02% and 37.75% of all 55,022 unigenes related to molecular function, respectively.

**Fig 4:**
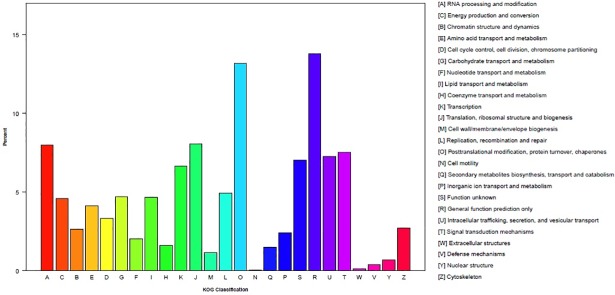
KOG functional classification of the sea buckthorn transcriptome. Note: 16,019 unigenes with significant homologies in the KOG database (E-value <10^−5^) were classified into 26 KOG categories. Capital letters on the x-axis indicate KOG categories on the right side of the histogram; the y-axis indicates the percentage of unigenes.

Within the sea buckthorn unigenes, 16,019(13.04%) were categorized (E-value <10^−5^) in 26 KOG clusters (Fig 4). The five largest categories were general function prediction only (2,211 genes, 13.8%); posttranslational modification, protein turnover, and chaperones (2,113 genes, 13.19%); translation, ribosomal structure and biogenesis (1,292 genes, 8.07%); RNA processing and modification (1,279 genes, 7.98%); and signal transduction mechanisms (1,162 genes, 7.52%).

### Metabolic pathway analysis by KEGG

**Fig 5:**
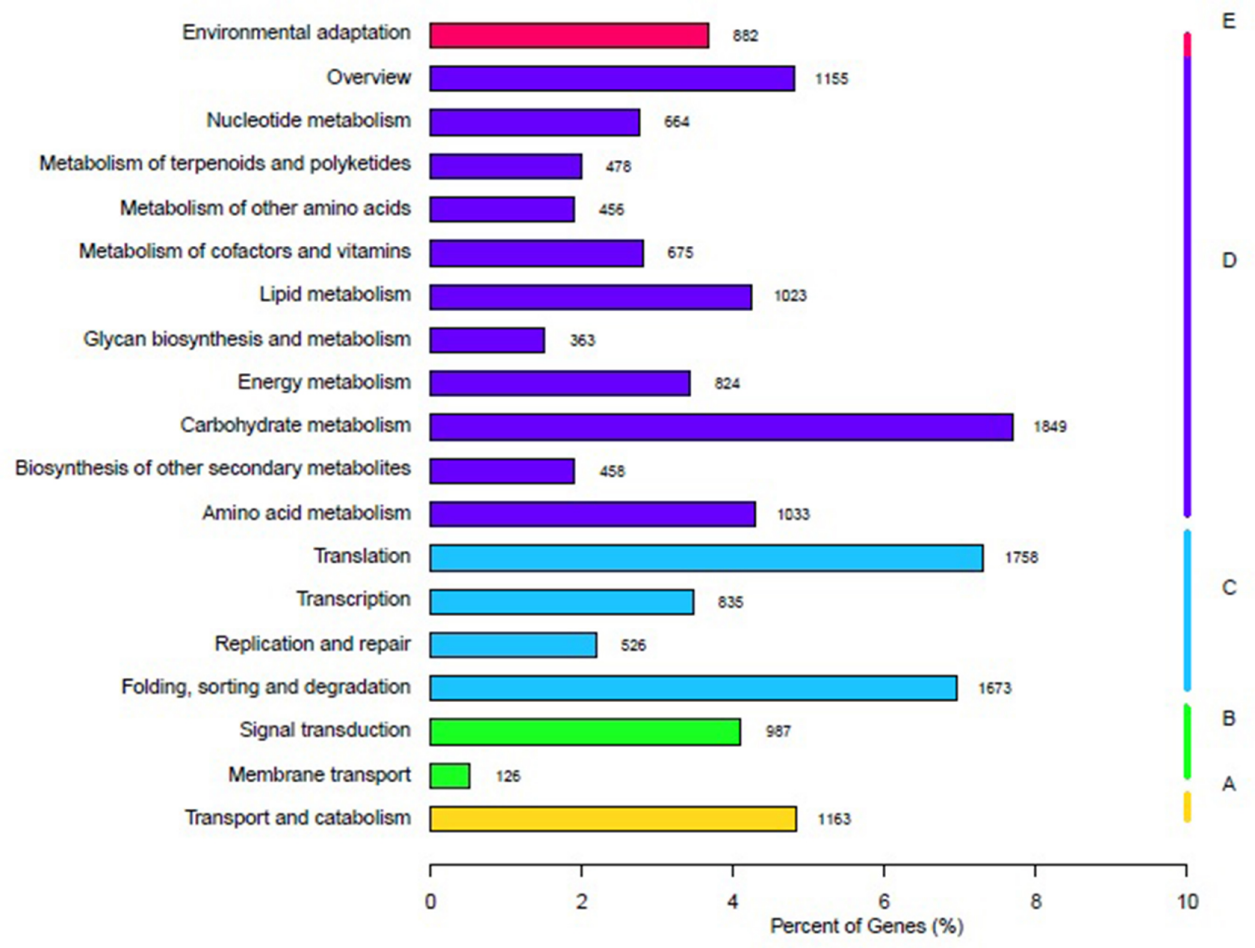
KEGG pathway annotation of sea buckthorn. Note: The percent of unigenes in each pathway is shown on the x-axis; the pathway categories are shown on the y-axis.

A total of 24,060 (19.59%) of the 122,803 unigenes from sea buckthorn had significant matches in KO. These unigenes were assigned to 19 KEGG pathways and can be divided into 5 groups (Fig 5). Among the KEGG pathways, 8,978 unigenes belonged to the largest group, metabolism (D), 4,792 belonged to genetic information processing (C), 1,163 belonged to cellular processes (A), 1,113 belonged to environmental information (B), and 882 belonged to organismal systems (E).

### CDS prediction

A total of 54,201 unigene CDSs were identified by the BLASTx protein database (NR and SwissProt database), of which 35,252 unigenes were longer than 500 bp, 20,539 unigenes were longer than 1,000 bp and 5,739 unigenes were longer than 2,000 bp. For another 43,713 unigenes that could not be identified by NR and SwissProt databases, we used Estscan (3.0.3) software to predict their ORF, including the length frequency distributions of unigenes CDSs and their corresponding amino acid sequences.

### Differentially expressed genes (DEGs) analysis

**Fig 6:**
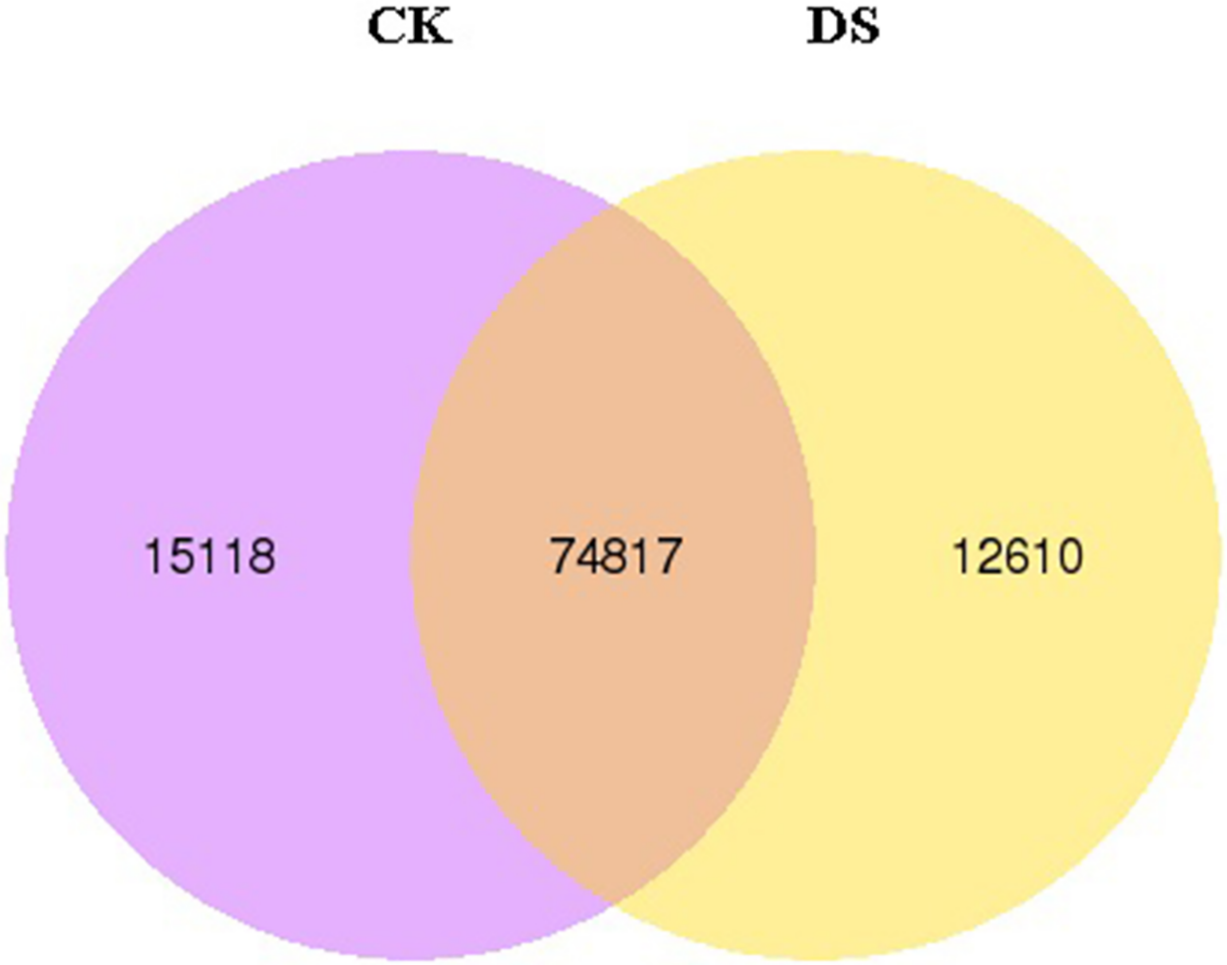
Venn diagram of specific and common DEGs in sea buckthorn. Note: The numbers of specific expressed and overlapped unigenes in CK and DS are shown in the Venn diagram.

**Fig 7:**
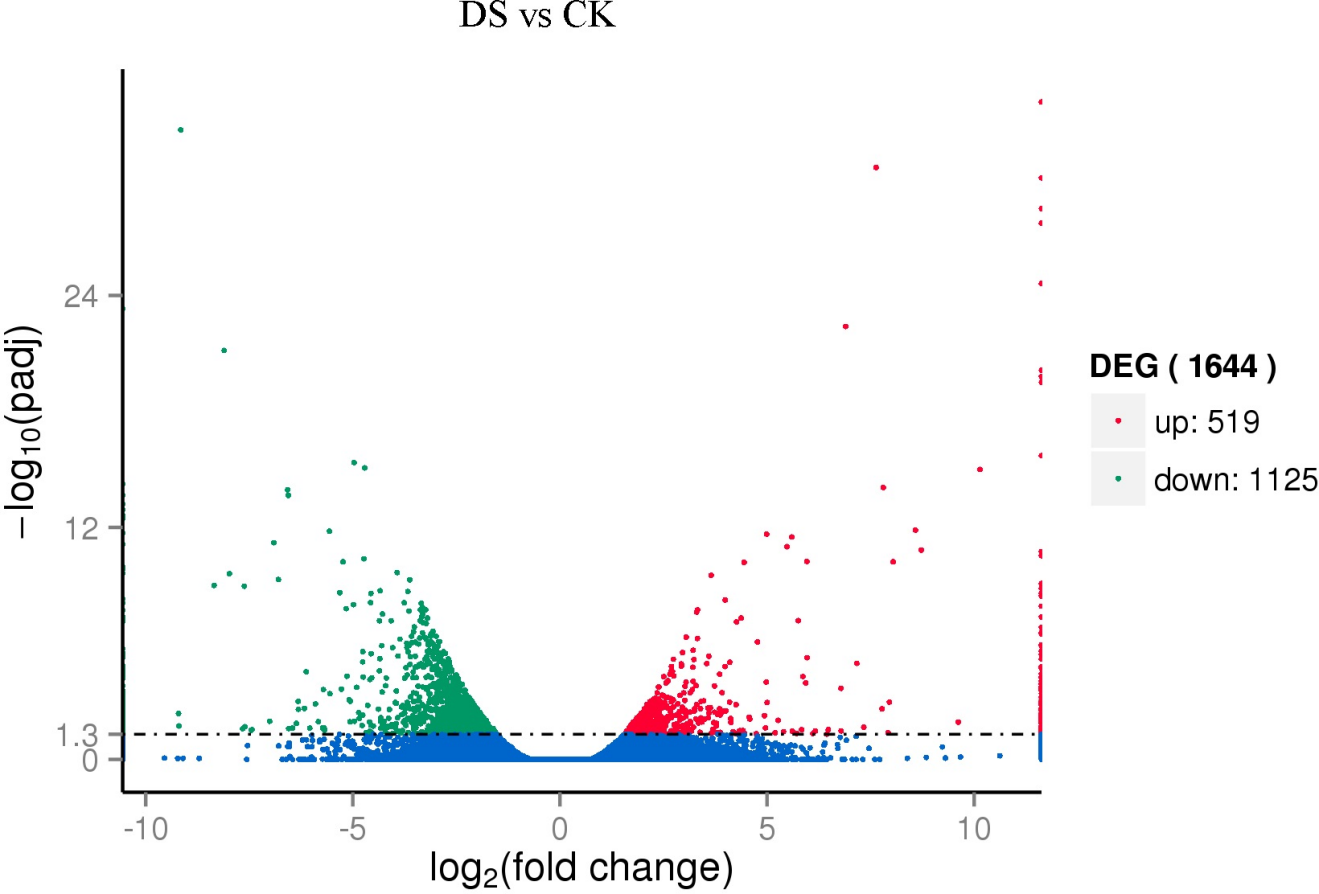
Volcano plot of differentially expressed unigenes of sea buckthorn. Note: The numbers of up- and down-regulated unigenes in CK and DS are shown. Red indicates up-regulated genes, green represents down-regulated unigenes, and blue represents no significant difference in expression.

Expression analysis of CK and DS showed that in the total 102,545 expressed unigenes (FPKM > 0.3), 74,817 were identified in both groups, with only 12,610 identified in drought stress-treated samples and only 15,118 identified in CK samples (Fig 6). Among these differentially expressed unigenes, the expression levels of 1,644 differed significantly between drought stress-treated samples (DS) and CK samples, of which 519 were up-regulated and 1,125 were down-regulated under drought stress (padj<0.05) (Fig 7). We also found that in these differentially expressed unigenes, only 129 were expressed after drought stress, and only 126 were expressed in CK, with no expression after drought stress.

### GO enrichment analysis

**Fig 8:**
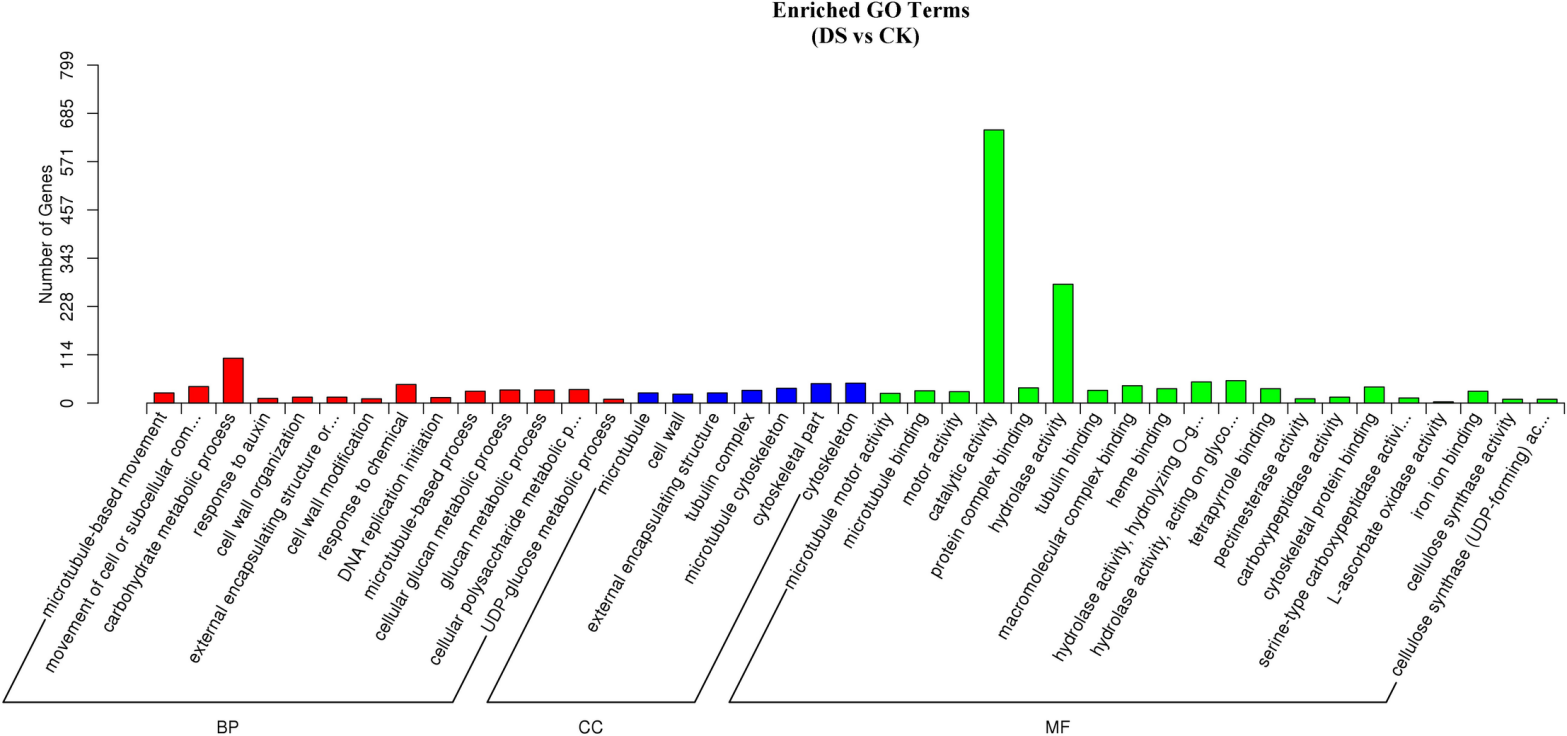
GO-enriched DEGs in sea buckthorn. Note: GO enrichment items of DEGs comparing DS with CK are shown. The graph only shows the top 20 items from each category; if the number of significantly enriched items was less than 20, all items are shown.

Based on GO functional annotations and enrichment analysis of the DEGs in sea buckthorn, 1142 DEGs were classified into three GO categories and 2439 items comparing DS with CK (some DEGs annotated in multiple terms), in which 1449 items were related to BP, 329 items were associated with CC and 661 items were associated with MF. The top 20 items significantly enriched in 3 GO categories are shown in Fig 8 (GO enrichment up-regulated and down-regulated items comparing DS with CK are shown in S1 Fig). In the 47 significantly enriched items (Corrected P-Value<0.05) 14 items were related to BP [microtubule-based movement (GO:0007018), movement of cell or subcellular component (GO:0006928), carbohydrate metabolic process (GO:0005975), response to auxin (GO:0009733), cell wall organization (GO:0071555), external encapsulating structure organization (GO:0045229), cell wall modification (GO:0042545), response to chemicals (GO:0042221), DNA replication initiation (GO:0006270), microtubule-based processes (GO:0007017), the cellular glucan metabolic process (GO:0006073), the glucan metabolic process (GO:0044042), the cellular polysaccharide metabolic process (GO:0044264), and the UDP-glucose metabolic process (GO:0006011)], 7 items were related to CC [microtubule (GO:0005874), cell wall (GO:0005618), external encapsulating structure (GO:0030312), tubulin complex (GO:0045298), microtubule cytoskeleton (GO:0015630), cytoskeletal part (GO:0044430), and cytoskeleton (GO:0005856)] and 26 items were related to MF [microtubule motor activity (GO:0003777), microtubule binding (GO:0008017), motor activity (GO:0003774), catalytic activity (GO:0003824), protein complex binding (GO:0032403), hydrolase activity (GO:0016787), tubulin binding (GO:0015631), macromolecular complex binding (GO:0044877), heme binding (GO:00200 37), hydrolase activity, hydrolyzing O-glycosyl compounds (GO:0004553), hydrolase activity, acting on glycosyl bonds (GO:0016798), tetrapyrrole binding (GO:0046906), pectinesterase activity, carboxypeptidase activity, cytoskeletal protein binding (GO:0008092), serine-type carboxypeptidase activity (GO:0004185), L-ascorbate oxidase activity (GO:0008447), iron ion binding (GO:0005506), cellulose synthase activity (GO:0016759), cellulose synthase (UDP-forming) activity (GO:0016760), xyloglucan: xyloglucosyl transferase activity (GO:0016762), nicotinate-nucleotide diphosphorylase (carboxylating) activity (GO:0004514), serine-type peptidase activity (GO:0008236), serine hydrolase activity (GO:0017171), oxidoreductase activity, acting on paired donors, with incorporation or reduction of molecular oxygen (GO:0016705), and ribonuclease T2 activity (GO:0033897)] and were significantly enriched under drought stress in sea buckthorn.

### KEGG pathway enrichment analysis

**Fig 9:**
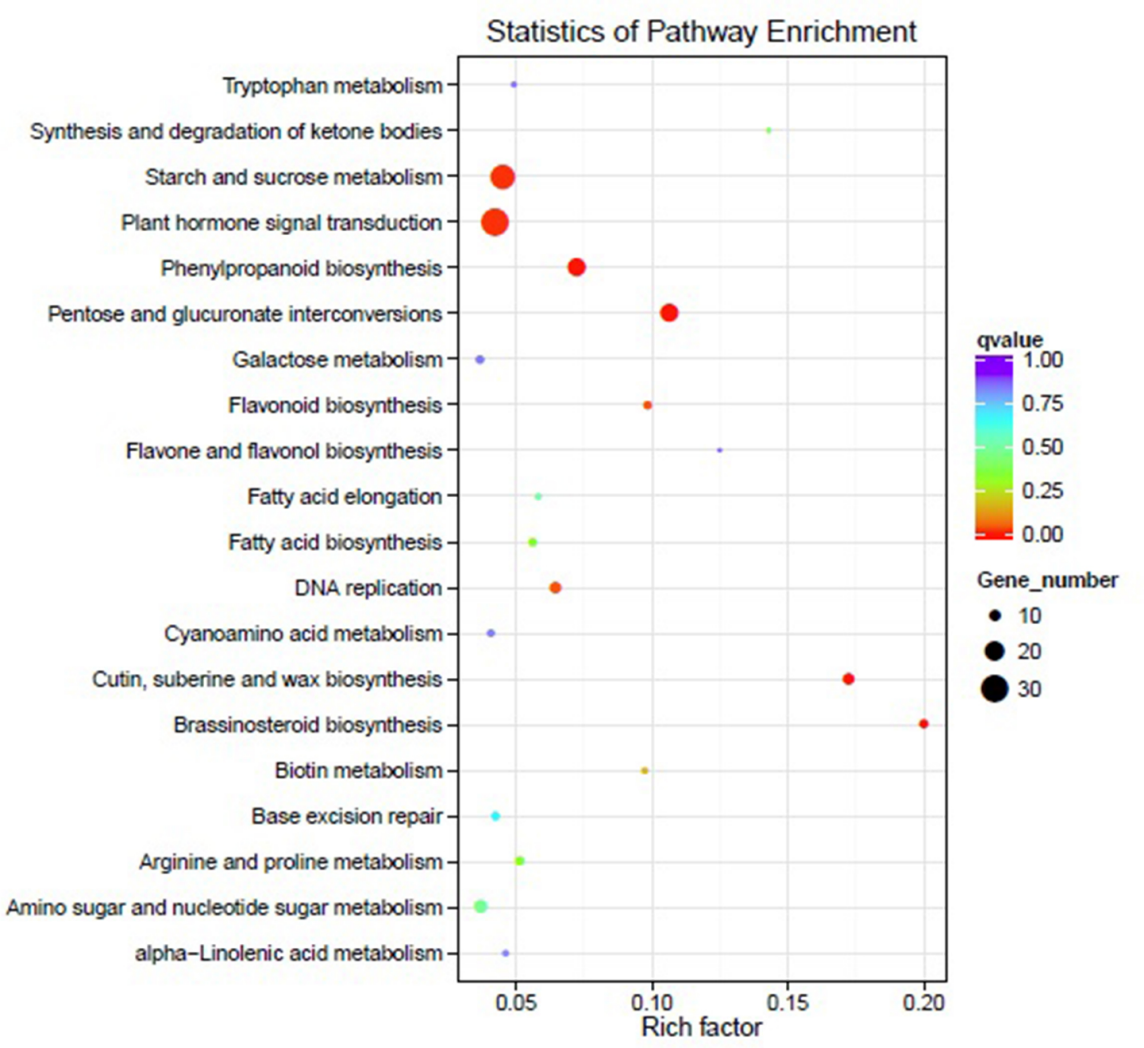
KEGG pathway enrichment of DEGs in sea buckthorn. Note: The graph shows only the top 20 enriched pathways comparing DS with CK; different colors denote different Q-Values, and the size of the bubble represents the number of DEGs.

According to the KEGG pathway enrichment analysis of the DEGs, there were 398 DEGs involved in 97 different pathways in sea buckthorn when comparing DS with CK (some DEGs were related to more than one pathway), in which 119 DEGs were up-regulated and 279 DEGs were down-regulated under drought stress. The top 20 significantly enriched pathways comparing DS with CK is shown in Fig 9. In these pathways, the significantly enriched pathways were pentose and glucuronate interconversions (18 unigenes), cutin, suberine and wax biosynthesis (10 unigenes), phenylpropanoid biosynthesis (18 unigenes), brassinosteroid biosynthesis (7 unigenes), starch and sucrose metabolism (26 unigenes), plant hormone signal transduction (30 unigenes), flavonoid biosynthesis (6 unigenes), and DNA replication (10 unigenes).

### Differentially expressed transcription factors (TFs) under drought stress

TFs play important roles in plant during the growth and development stage and can activate or inhibit gene expression at the transcriptional level to help plants maintain normal physiological activity under stress. In this paper, based on iTAK, we found a total of 4438 TFs in sea buckthorn, including 3682 TFs and 756 transcription regulatory factors, which belong to 80 and 23 families, respectively (S2 and S3 Tables). Differential analysis showed 100 TFs that were differentially expressed comparing DS with CK, of which 41 TFs were up-regulated and 59 TFs were down-regulated. These differentially expressed TFs belong to 25 TF families and 11 transcription regulatory factor families, respectively (Table 2). The fold changes of DEGs of all TFs below 4 were compared between DS and CK, except for 17 TFs that were only expressed in CK or DS.

**Table 2:** DEGs of Transcription factors and transcription regulatory factors of sea buckthorn.

TF families	Down- regulated	Up- regulated	TF families	Down- regulated	Up- regulated
ABI3VP1	1	0	NAC	1	4
AP2-EREBP	5	2	Orphans	1	2
ARF	1	0	PLATZ	0	2
bHLH	5	0	Pseudo ARR-B	0	1
bZIP	6	2	ARID	1	0
C2C2-Dof	1	0	AUX/IAA	3	1
C2C2-YABBY	1	0	GNAT	0	1
C2H2	0	1	HMG	4	0
C3H	1	1	TUB	1	0
CCAAT	2	0	WRKY	1	5
CPP	4	0	Jumonji	0	1
E2F-DP	1	0	LIM	1	0
FHA	1	0	mTERF	1	1
G2-like	0	1	PHD	1	0
GRAS	1	1	SET	3	1
HB	0	5	SNF2	1	1
HSF	0	1	SWI/SNF-BAF60b	1	0
MYB	8	6	TRAF	1	1

### Validation of DEGs from RNA-Seq

**Fig 10:**
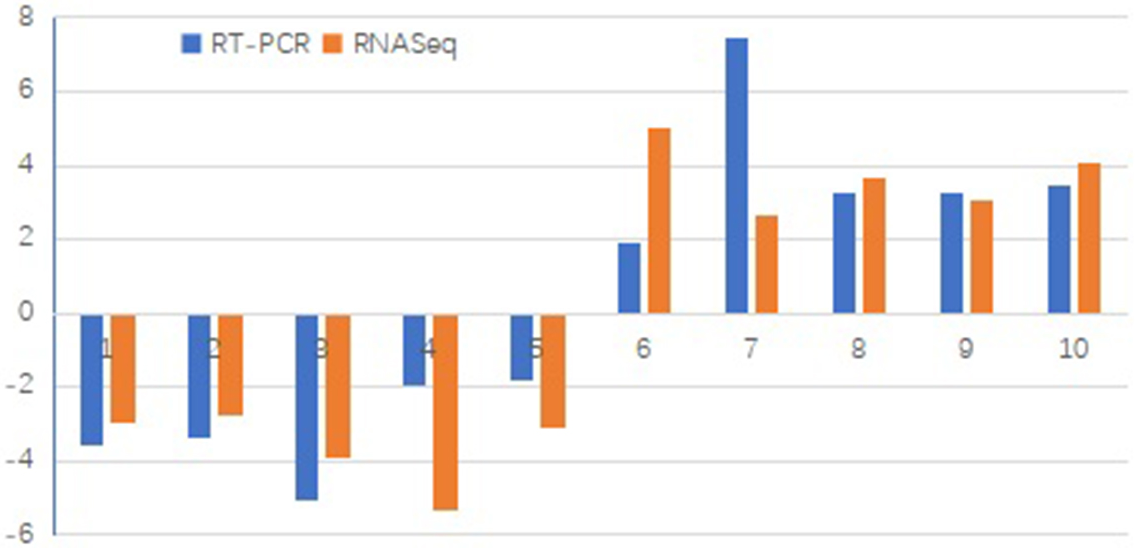
Validation of DEGs by qRT-PCR. Note: qRT-PCR values were calculated as the means from sea buckthorn under CK and DS. The x-axis indicates the different DEGs, and the y-axis indicates the relative expression level, calculated as log2 (change fold).

To validate the RNA-Seq gene expression results, quantitative reverse transcription-PCR (qRT-PCR) was performed to assess the expression levels of 10 randomly selected DEGs of sea buckthorn under control and drought stress conditions. Fig 10 compares the expression of RNA-Seq and qRT-PCR, and although there is some difference in the absolute fold change, their expression trends were largely consistent between the two methods among the 10 DEGs.

## Discussion

Because of their sessile lifestyle, plants often encounter unfavorable environmental conditions such as drought, flood, salt, heat and cold. Drought stress is one of the most significant environmental factors constraining crop production around the world. To enhance resistance to drought stress and to breed drought stress-resistant crops, many scientists have endeavored to clarify the mechanisms of stress responses in plants according to various aspects. Plants’ responses to abiotic stress are well-known, but the drought stress response is still a complex phenomenon with several key factors that have yet to be investigated.

To survive under harsh environmental conditions, plants have evolved an intricate system through a long evolutionary progress at multiple levels to perceive external signals and transduce the stress signal in a timely fashion, leading to a series of reactions at different levels, e.g., morphological, physiological and biochemical and molecular levels [40,41].

### Morphological characteristics related to drought stress

As a strong drought stress-resistant plant, sea buckthorn has evolved a series of morphological characteristics to adapt to drought stress, such as a strong root system, lanceolate leaf, abundant thorns, epidermis covered with thickened cuticle and wax, and deeply sunken stomata covered by densely overlapped stellate hair and scales[42,43]. Drought stress can lead to metabolic changes in cutin and wax, which is eventually achieved through the regulation of related gene expression. In this study, one unigene related to the biosynthesis of wax (Cluster-17196.90766) was up-regulated at the transcript level and enriched under DS compared with CK. As important morphological specificities of drought stress resistance, cutin and wax play an important role during drought stress by preventing water loss through nonstomatal transpiration, thus improving water use efficiency in plants. Our result indicating that it participates in the process of the adaptation of sea buckthorn to drought stress.

### ABA signaling and regulation in response to drought stress

Under drought stress, plants will perceive stress first and then trigger a series of signal transduction cascades with the pathways transduced by phytohormones[44,45], therefore plant hormones plays very important roles in response to drought stresses, and among the phytohormones, ABA is considered a major regulator under abiotic stresses. Under drought stress, the content of ABA in plants will increase and will lead to stomatal closure to prevent water loss through transpiration through stomata. In our results, we found 18 unigenes that encode NCED (9-cis-epoxycarotenoid dioxygenase) in sea buckthorn, the key enzyme responsible for ABA biosynthesis. Of these, 15 are up-regulated, but none showed expression that was significantly different between DS and CK, possibly because under stress, sea buckthorn roots will perceive stress first and then produce ABA, which will translocate to the above-ground parts through the stem transportation system and trigger short-term responses including stomatal closure[46,47], which will help sea buckthorn to prevent water loss and enhance water retention capacity under water deficit status. Therefore, perception of the stress signal and subsequent molecular signaling in sea buckthorn roots must be clarified in a subsequent study to uncover the drought stress resistance mechanism.

When plants are exposed to drought, the increase in ABA levels results in binding to PYR/PYL, which alters the conformation of the PYR/PYL protein, and this change allows PYR/PYL to interact with the negative regulator type 2 C protein phosphatase (PP2C) to form a temporary complex (ABA-PYR/PYL-PP2C) that can inhibit PP2C activity and activate the positive regulator SNF1-related protein kinase 2 (SnRK2s), whose kinase activity is inhibited by PP2Cs. This will induce the expression of downstream stress-responsive genes and allow plants to adapt to a water shortage environment[48]. In our results, we identified 269 PP2C, of which 1 gene was notably down-regulated (Cluster-17196.15336(-2.2994)), and 57 SnRK2 were identified, of which 3 was significantly up-regulated under drought stress (Cluster-17196.82913 (2.101), Cluster-17196.82914 (2.6943), and Cluster-17196.33864 (2.839)). This indicates that under drought stress, the lower expression of PP2C in sea buckthorn will release the inhibition of SnRK, and its expression will induce stomatal closure and activate the downstream drought stress-related genes and help sea buckthorn maintain normal growth and survival under drought conditions.

### The genes and functional proteins responsive to drought stress

In plants, the genes responsive to unfavorable conditions can easily be divided into two groups [17]: one is regulatory proteins, such as transcriptional factors, protein kinase, phosphatase and phospholipid metabolic enzymes, which can adjust stress-related gene expression under abiotic stress. The other group is functional proteins, including small molecular osmotica (e.g., proline, betaine, and soluble sugar), LEA (later embryogenesis abundant), protect enzymes (e.g., SOD, CAT, and POD), and water channel proteins. These proteins will prevent the cells of plants that encounter stress from damage by maintaining turgor pressure, as well as through oxygen free radical scavenging and protection of the structure of intracellular biomacromolecules [49]TFs can activate or inhibit the transcriptional expression of downstream stress-related genes under water deficit conditions. It has been confirmed that members of the transcription factor family such as AP2, bHLH, bZIP, C2H2, HB, NAC, DREB, MYB, PLATZ and WRKY are involved in plant response to abiotic stress[50,51]. In our research, we found 4438 TFs in sea buckthorn, but we found only 98 DEGs, and among them, 40 were up-regulated and 58 were down-regulated. We can easily see that a large amount of TF expression is not altered significantly under drought stress. In the DEGs, ABI3VP1, ARF, C2C2-Dof, C2C2-YABBY, CCAAT, CPP, E2F-DP, FHA, ARID, TUB, LIM PHD and SWI/SNF-BAF60b are all down-regulated, and C2H2, G2-like, HB, HSF, GNAT, PLATZ, Pseudo ARR-B,WRKY and Jumonji are all up-regulated. For other TF families such as AP2-EREBP, bZIP, C3H, GRAS, MYB, NAC, Orphans, AUX/IAA, mTERF, SET, SNF2 and TRAF, some members are up-regulated and some are down-regulated. Different genes belonging to the same family showed different expression patterns under drought stress, which may indicate a different function in stress response, and their response to water deficit conditions can be positive or negative[52]. A single transcription factor could interact with one or more other members in the same family and even other families[53], which also shows the intricacy by which the transcriptional family of genes regulates the water shortage response in sea buckthorn. All the results provide valuable information for further functional analyses of these TFs’ functions in drought stress resistance in sea buckthorn.

Protein phosphorylation and dephosphorylation are one of the most important post-translational protein modifications during signal transduction in plants under abiotic stress[54,55], Many regulatory proteins and enzymes can be switched on and off by phosphorylation and dephosphorylation to control a wide range of cellular processes or signal relays. It is thus possible to freeze or activate the enzyme under stress conditions without changing the concentration of intracellular enzymes or related proteins, and it has been confirmed that protein phosphorylation modification plays an important role in the response to stress[54,56]. It’s reported that there are more than 1000 kinases in the Arabidopsis genome [57], in which MAPK (Mitogen-activated protein kinase) and CDPK (calcium-dependent protein kinase) are two of the most important signaling pathways in plant under abiotic stress. The MAPK cascade can transduce extracellular signals into cellular responses through sequential phosphorylation, which leads to phosphorylation of other downstream proteins to activate or repress their functions [58]. As a special sensor, CDPKs can directly convert upstream Ca^2+^ signals into downstream protein phosphorylation events due to its multifunctional protein structure, which combines calcium-binding and signaling capabilities within a single gene product [59]. In our results, we found 3539 protein kinases, which belong to 15 protein kinase families. Of these, we identified 92 DEGs, among which 67 unigenes were down-regulated and 25 unigenes were up-regulated, in addition, 122 unigenes coding MAPK and 54 unigenes coding CDPK were identified, and there are 6 DEGs (3 up- and 3 down-regulated) and 1 DEG (down-regulated), respectively. This indicates that the MAPK and CDPK pathways participate in drought stress signaling as signal transduction factors and may play a crucial role in osmotic stress responses in sea buckthorn.

When plants are involved in drought stress conditions, osmotic stress will induce the accumulation of ROS (reactive oxygen species) in cells, subjecting the plant to oxidative stress. Plants have already evolved an antioxidant defensive system to scavenge ROS and mitigate cellular damage; this system includes SOD, POD and CAT. In this paper, we found 21 SODs (belonging to 3 families), 7 CATs and 148 PODs. For SOD and CAT, although most are up-regulated, there are no significantly expressed genes, and the 10 DEGs of POD are all down-regulated. This may be because we only sampled two experimental points (control and drought stress treatment) and because the expression of protective enzymes is a dynamic process. At the beginning of drought stress, their expression will be enhanced, and with the increase in stress treatment time, their expression should be decreased. Further study is needed to identify the expression mode of these enzymes over different stress time periods.

In addition, small-molecule proteins such as LEA (late embryogenesis abundant), proline, and betaine also play important roles in preventing cells from the harmful effects of unfavorable and extreme conditions. As a very important osmotic adjustment, LEA is a large protein family that was first shown to accumulate during seed desiccation in the later stages of embryogenesis. In this paper, we identified 47 unigenes that may code LEA. There are 4 DEGs, all of which were up-regulated comparing DS with CK in sea buckthorn. Thus, these unigenes are essential for the resistance to water shortage for sea buckthorn.

In addition to osmotic regulation, the ability to prevent moisture loss is another key figure for plants to endure stress under a dehydrated environment. Plants have a main continuous water channel system that is responsible for the transmembrane bidirectional flux of water and long-distance water transportation, which is known as AQP (aquaporin) and located in the plasma membranes and tonoplast membranes in leaves and the epidermal and inner cells of the root and vascular tissues of stems. It is confirmed that plants can resist various abiotic stresses by controlling the activity of water channel proteins; in sea buckthorn, we found 92 unigenes with the code AQP, with 9 unigenes differentially expressed, 8 sharply down-regulated and only 1 up-regulated. The expression of AQPs decreased at the transcriptional level indicating that the activity of AQPs decreased or even disappeared, and the closure of AQPs can restrict water loss and maintain water balance in plant cells, thus increasing tolerance to drought stress in sea buckthorn. For the 1 AQP whose expression increased under drought stress, further research is required to understand the spatial expression under dehydration and clarify its function in response to drought stress. Fatty acid and starch metabolism under drought stress.

To survive in harsh environments—in addition to a response to stress through signal regulation and functional gene expression—plants also alter the synthesis and catabolism of macromolecules such as fatty acids, carbohydrates, and proteins to reserve energy sources as a prolonged energy supply to maintain cell survival [60]. In our results, the pathway of fatty acid degradation (ko00071) is up-regulated, and pathways such as the biosynthesis of unsaturated fatty acids (ko01040), fatty acid elongation (ko00062), and starch and sucrose metabolism (ko00500) are down-regulated. All of these pathways are significantly enriched under drought stress. The degradation of fatty acid and starch will increase hexose levels in the cell and provide energy for sea buckthorn under stress.

## Conclusion

Under water deficit stress, stress signal perception, signal transduction, expression of the regulatory genes and the corresponding downstream functional genes are the key factors in plant response to adversity. In this research, based on RNA-Seq, we identified 122,803 unigenes in sea buckthorn, in which 1,644 unigenes were differentially expressed between DS and CK, of which 519 unigenes were up-regulated and 1,125 unigenes down-regulated. In addition, we found 4438 transcriptor factors (TFs) in sea buckthorn, of which 100 were differentially expressed between DS and CK. The results suggest that these genes are involved in and have a highly important and complicated role in the drought stress resistance process. This work lays the first foundation for further investigations of the specific functions of these genes in sea buckthorn under drought stress and other abiotic stress factors. It also provides candidate genes for engineering plants to promote drought stress resistance.

## Supporting information

S1 Fig GO enrichment items of up-regulated and down-regulated genes comparing DS with CK. Fig A: GO enrichment items of up-regulated genes comparing DS with CK. Fig B: GO enrichment items of down-regulated genes comparing DS with CK.
(TIF)

S1 Table Primer information used for quantitative PCR analysis. (DOCX)

S2 Table Transcription factor families of sea buckthorn. (DOCX)

S3 Table Transcription regulatory factor families of sea buckthorn. (DOCX)
