# Supplementary figures and images for: Transcriptomic analysis of drought stress responses of sea buckthorn (Hippophae rhamnoidessubsp. sinensis) by RNA-Seq
Source: PLoS One. 2018 Aug 13;13(8):e0202213. doi: 10.1371/journal.pone.0202213 (PMC6089444; doi:10.1371/journal.pone.0202213)

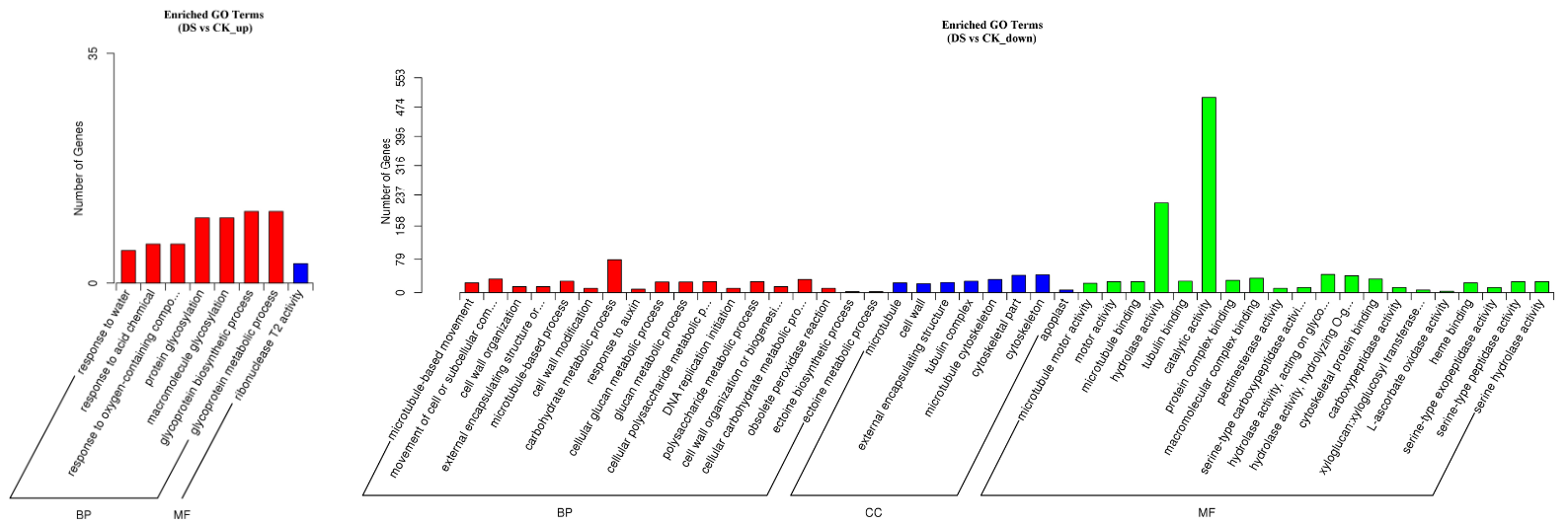

Supplement: S1 Fig — Fig A: GO enrichment items of up-regulated genes comparing DS with CK. Fig B: GO enrichment items of down-regulated genes comparing DS with CK. (TIF) [file pone.0202213.s001.tif]
