# Supplementary material for: Transcriptomic analysis of drought stress responses of sea buckthorn (Hippophae rhamnoidessubsp. sinensis) by RNA-Seq
Source: PLoS One. 2018 Aug 13;13(8):e0202213. doi: 10.1371/journal.pone.0202213 (PMC6089444; doi:10.1371/journal.pone.0202213)
Supplement: S1 Table — (DOCX) [file pone.0202213.s002.docx]

**S1 Table| Primer information used for Quantitative PCR analysis**

| Unigene ID | Primer sequences | Gene name |
| --- | --- | --- |
| Cluster-17196.19201 | 5’- ACGAGAGACAGGGAACGAGTG-3’ | BUBR1 |
|  | 5’- TTCTTGAGGTGATTGTGGTTATGAG-3’ |  |
| Cluster-17196.70802 | 5’- GGTCACATGGAACATACTGGAAA-3’ | Tyrosine hydroxylase/AT1G16860 |
|  | 5’-AACCCACAAGCAAACCCATC -3’ |  |
| Cluster-17196.96726 | 5’- TCACTCATAGCTGAGACCTTTTCTG-3’ | ATHB-12 |
|  | 5’-ACCTTCGTTTGTTCTTGCTCTTCT -3’ |  |
| Cluster-17196.50084 | 5’-GCTGGTTGTTGTGGTGCTGT -3’ | Plastocyanin-like domain |
|  | 5’- CCCTCACTGGTTGGACTTGG-3’ |  |
| Cluster-17196.55680 | 5’-CATTCGCTTGGAGGAGGAAC -3’ | TUBB |
|  | 5’-CTTGGGTGATGGGAAAACAGA -3’ |  |
| Cluster-17196.84715 | 5’- AAGGGATTGGGAGGCAGAA-3’ | IMK |
|  | 5’-AGGAAGGTATCCTAATGCTGATGG -3’ |  |
| Cluster-4109.3 | 5’-ATTGGTTGTCCGCCTTGC -3’ | RNA-binding protein |
|  | 5’-TGTGCTTGTTTGGCTTGCTT -3’ |  |
| Cluster-17196.97436 | 5’-TGATTTCTGTCTTGGCTAGTGGTT -3’ | GALT1 |
|  | 5’- TGGTGTTTTGGGAGAGAGAGG-3’ |  |
| Cluster-17196.97600 | 5’-TCAAAAGGACTGCAATGGAAAG -3’ | uncharacterized protein LOC103336914 isoform X1 |
|  | 5’-GCCCGCAGATAGCACAAAC -3’ |  |
| Cluster-17196.42957 | 5’- CATCTTTGTCCTGCTTCATCTTGT-3’ | PCNA |
|  | 5’- AGGGAATAATCCTTTACCCCACA-3’ |  |
| Reference gene | 5’-CACGAGACCACATACAACTCCA-3’ | β-actin |
|  | 5’-TTGAACCACCACTGAGAACGA-3’ |  |
