# Supplementary material for: Transcriptomic analysis of drought stress responses of sea buckthorn (Hippophae rhamnoidessubsp. sinensis) by RNA-Seq
Source: PLoS One. 2018 Aug 13;13(8):e0202213. doi: 10.1371/journal.pone.0202213 (PMC6089444; doi:10.1371/journal.pone.0202213)
Supplement: S2 Table — (DOCX) [file pone.0202213.s003.docx]

**S2 Table| Transcription factor families of sea buckthorn**

| **TF families** | **Number** | **TF families** | **Number** | **TF families** | **Number** |
| --- | --- | --- | --- | --- | --- |
| **ABI3VP1** | 68 | CSD | 6 | Orphans | 205 |
| **Alfin-like** | 13 | DBP | 4 | PBF-2-like | 2 |
| **AP2-EREBP** | 213 | E2F-DP | 24 | PLATZ | 24 |
| **ARF** | 75 | EIL | 22 | RWP-RK | 16 |
| **ARR-B** | 13 | FAR1 | 79 | S1Fa-like | 1 |
| **BBR/BPC** | 21 | FHA | 64 | SAP | 3 |
| **BES1** | 11 | G2-like | 103 | SBP | 73 |
| **bHLH** | 238 | GRAS | 93 | SET | 123 |
| **BSD** | 18 | GRF | 29 | Sigma 70-like | 7 |
| **bZIP** | 174 | GeBP | 9 | SRS | 18 |
| **C2C2-CO-like** | 9 | HB | 198 | TCP | 35 |
| **C2C2-Dof** | 70 | HRT | 1 | TUB | 20 |
| **C2C2-GATA** | 60 | HSF | 65 | Tify | 26 |
| **C2C2-YABBY** | 10 | LOB | 48 | TIG | 10 |
| **C2H2** | 142 | MADS | 74 | Trihelix | 53 |
| **C3H** | 190 | MYB | 341 | ULT | 3 |
| **CAMTA** | 10 | mTERF | 69 | VOZ | 4 |
| **CCAAT** | 88 | NAC | 177 | WRKY | 161 |
| **CPP** | 14 | OFP | 24 | Zf-HD | 31 |
