# Supplementary material for: Transcriptomic analysis of drought stress responses of sea buckthorn (Hippophae rhamnoidessubsp. sinensis) by RNA-Seq
Source: PLoS One. 2018 Aug 13;13(8):e0202213. doi: 10.1371/journal.pone.0202213 (PMC6089444; doi:10.1371/journal.pone.0202213)
Supplement: S3 Table — (DOCX) [file pone.0202213.s004.docx]

**S3 Table| Transcription regulatory factor families of sea buckthorn**

| **Transcription regulatory factors families** | **Number** | **Transcription regulatory factors families** | **Number** |
| --- | --- | --- | --- |
| ARID | 48 | MED7 | 4 |
| AUX/IAA | 86 | PHD | 109 |
| Coactivator P15 | 4 | Pseudo ARR-B | 8 |
| DDT | 17 | RB | 7 |
| GNAT | 101 | Rcd1-like | 15 |
| HMG | 14 | SNF2 | 85 |
| IWS1 | 27 | SOH1 | 3 |
| Jumonji | 53 | SWI/SNF-BAF60b | 60 |
| LIM | 2 | SWI/SNF-SWI3 | 14 |
| LUG | 8 | TAZ | 17 |
| MBF1 | 6 | TRAF | 66 |
| MED6 | 2 | Total | 756 |
